# Simian Foamy Virus in Non-Human Primates and Cross-Species Transmission to Humans in Gabon: An Emerging Zoonotic Disease in Central Africa?

**DOI:** 10.3390/v5061536

**Published:** 2013-06-19

**Authors:** Augustin Mouinga-Ondémé, Mirdad Kazanji

**Affiliations:** 1 Unité de Rétrovirologie, Centre International de Recherches Médicales de Franceville (CIRMF), Franceville BP 769, Gabon; E-Mail: ondeme@yahoo.fr; 2 Institut Pasteur de Bangui, Bangui BP 923, Central African Republic

**Keywords:** SFV, mandrills, wild-born non-human primates, interspecies transmission, Gabon, central Africa

## Abstract

It is now known that all human retroviruses have a non-human primate counterpart. It has been reported that the presence of these retroviruses in humans is the result of interspecies transmission. Several authors have described the passage of a simian retrovirus, simian foamy virus (SFV), from primates to humans. To better understand this retroviral “zoonosis” in natural settings, we evaluated the presence of SFV in both captive and wild non-human primates and in humans at high risk, such as hunters and people bitten by a non-human primate, in Gabon, central Africa. A high prevalence of SFV was found in blood samples from non-human primates and in bush meat collected across the country. Mandrills were found to be highly infected with two distinct strains of SFV, depending on their geographical location. Furthermore, samples collected from hunters and non-human primate laboratory workers showed clear, extensive cross-species transmission of SFV. People who had been bitten by mandrills, gorillas and chimpanzees had persistent SFV infection with low genetic drift. Thus, SFV is presumed to be transmitted from non-human primates mainly through severe bites, involving contact between infected saliva and blood. In this review, we summarize and discuss our five-year observations on the prevalence and dissemination of SFV in humans and non-human primates in Gabon.

## 1. Introduction

Foamy viruses are members of the *Spumavirus* genus of the Retroviridae family [1]. These exogenous complex retroviruses are highly prevalent in several animal species, including felines, bovines and equines, in which they cause persistent infections [2,3,4,5,6]. They are also highly prevalent in several species of non-human primate [7], the documented level of infection being 75%–100% in captive adults, especially macaques and baboons [8,9]. Recently, it was shown that non-human primates living in the wild, comprising various species of monkey and ape in Africa (mandrills, gorillas, chimpanzees) and Asia (species of macaques), also have a high prevalence of simian foamy virus (SFV) infection [10,11,12].

Infection with SFV has been reported in 1%–4% of people occupationally exposed to non-human primates in zoos, primate centres and laboratories, mainly in North America but also in Europe [13,14,15,16,17]. Naturally acquired SFV infections have also been described in hunters in central Africa [18,19,20,21,22] and in several people with frequent contact with macaque species in Asia [23]. 

SFV is presumed to be transmitted from non-human primates to humans mainly through severe bites, thus involving contact between the virus present in the oral mucosa and the blood of bitten individuals [8,9,24]. It has been shown previously that bites by adult non-human primates are the major risk factor for viral acquisition in hunters and people exposed occupationally, such as zookeepers, veterinarians and personnel in animal care facilities [25,26]. 

Until now, infection-associated disease in humans and human-to-human transmission of SFV, has not been documented [19,25,26,27]. Foamy viruses have been considered to be non-pathogenic in naturally and experimentally infected animals [28,29] and did not appear to cause disease in the few accidentally infected humans who received long-term medical and biological follow-up [14,17,19,27,30,31]. This lack of pathogenicity contrasts strongly with the cytopathic effect seen *in vitro* in infected cell cultures, with the appearance of “foam-like” syncitia [28,32,33]. 

In contrast to HIV and SIV lentiviruses, foamy viruses show low genetic drift *in vivo* [2,9,34,35] and are considered to be genetically stable [36]. Phylogenetic analysis has demonstrated species-specific transmission of foamy viruses, indicating long-term co-evolution with their natural hosts, suggesting that foamy viruses have co-speciated in Old World primates for at least 30 million years [37]. Their high genome conservation often allows attribution to a particular monkey or ape subspecies by analysis of the appropriate foamy virus sequence [8,12,38]. Furthermore, in cross-species transmission to humans or apes, the transmitted virus can easily be traced back to the transmitting monkey species and appears to be genetically stable in the new host for decades [27,34,39]. Once acquired, SFV infections persist for life [3,9,35]. While the molecular features of foamy viruses have been studied extensively *in vitro* [28,32,33,40], little information is available on their characteristics *in vivo*, such as epidemiological determinants [3,9,10,29,34,41]. For example, little is known about the timing and modes of primary infection or about the chronic phase of infection, including the dynamics of viral load, viral expression and virus transmission. 

Gabon is located in central Africa and traversed by the equator; nearly 80% of the surface area of 267,667 km^2^ is covered by rainforest. The country has about 1.5 million inhabitants (5.6 inhabitants/km^2^), 73% of whom live in urban areas. Administratively, Gabon is divided into nine provinces with 2048 villages located mainly along roads and rivers; few have more than 300 inhabitants. The main activities are subsistence farming, hunting, gathering and fishing. A wide diversity of non-human primates live deep in the rainforest, and hunting monkeys and apes is still frequent, with wide circulation of “bush meat”.

In this review, we summarize observations made over five years on the prevalence and the dissemination of SFV in captive mandrills and wild non-human primates. We also present our data on the cross-species transmission of this virus to humans and discuss zoonotic infection in the human population and the potential risk for dissemination of the virus to humans living in this area of central Africa. 

## 2. SFV Infection in Mandrills

**Figure 1:**
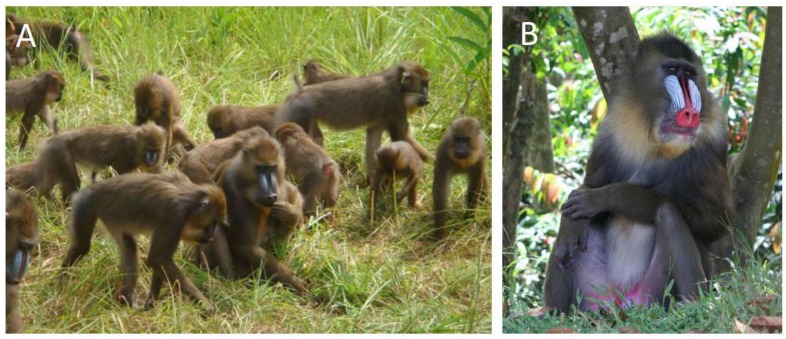
The mandrill colony at the International Centre for Medical Research (CIRMF) in Franceville, Gabon. (**A**) Semi-free-ranging colony of mandrills at the Primate Centre of the CIRMF; (**B**) adult male mandrill.

Mandrills (*Mandrillus sphinx*) (Figure 1) are found in the wild in a restricted area of central Africa, in the tropical forests of Cameroon, Equatorial Guinea, Gabon and southern Congo [42]. A semi-free-ranging colony of mandrills was created at the Primate Centre of the International Centre for Medical Research (CIRMF) in Gabon in 1983, and more than 140 mandrills are now housed on a 9.5-hectare enclosure [42]. It was reported previously that mandrills are naturally infected with SIV (SIVmnd) and simian T-cell leukaemia virus (STLV-1) [42,43,44,45,46,47,48,49], but little information is available on SFV infection in mandrills. Calattini *et al.* reported SFV infection in a small series of wild-born, wild-caught mandrills in Cameroon and five mandrills in the Primate Centre in Gabon [10]. Furthermore, interspecies transmission of SFV from mandrills to humans has been reported [18,19,50]. 

**Figure 2:**
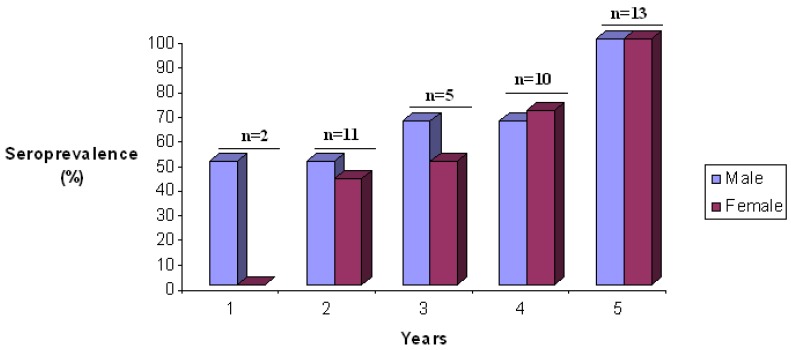
Seroprevalence of simian foamy virus (SFV) antibodies in young female and male semi-free-ranging mandrills at the CIRMF colony.

We evaluated the natural history of SFV in this mandrill colony, including the prevalence, modes of transmission, genetic diversity and origin. These studies received ethical approval from the Ministry of Health and from the Ethical Committee of the CIRMF. All human subjects received detailed information and gave consent during personal interviews [20]. We found by western blot analysis that 83% of the mandrills were SFV seropositive, and the seroprevalence increased significantly with age, from 57% in juvenile monkeys to 94% in adults and 100% in older mandrills (Figure 2). Seroconversion at an early age could be due to exchange of saliva between young mandrills and their mothers during feeding, as mandrills have a prominent muzzle–muzzle behaviour, usually between young naive and older individuals [51]. 

Seroprevalence increased with age, with no significant difference between males (84%) and females (82%). In previous studies on *Macaca tonkeana*, it was shown that the monkeys acquire SFV mainly through severe bites when young adults (5–8 years) compete for sexual partners [8]. Furthermore, in a study of free-ranging colonies of chimpanzees, Liu *et al.* found a significant increase in SFV infection with age, with no evidence of perinatal transmission [12]. These findings indicate that horizontal rather than vertical transmission is the predominant route of SFV infection in these non-human primate communities. Some species or colony specificity among troops of nonhuman primates may, however, change the relative importance of different modes and thus the timing of SFV transmission. 

To evaluate the SFV strains circulating in our mandrill colony, a portion of the *integrase* gene was amplified in DNA obtained from PBMCs of seropositive animals, and SFV was detected by PCR in 87% of the samples. A comparison of nucleotide sequences showed that the majority were closely related, with 94%–100% sequence similarity, and they were also closely related to the five SFV sequences obtained by Calattini *et al.* [10]. Some divergent samples were obtained, however, from a wild-born mandrill introduced into the colony at the age of two years, which showed greater nucleotide divergence (8%–9%) than all the other mandrill SFV sequences. As this animal was born in the wild, it might have been infected before introduction into our colony. This virus was more divergent than those found in the colony, and we hypothesize that various stains of SFV can infect mandrills. 

**Figure 3:**
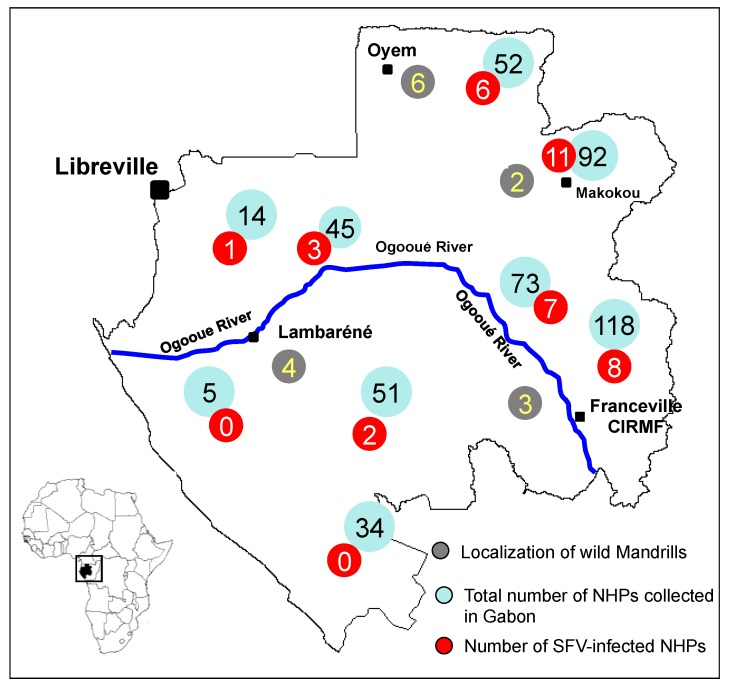
Map of Gabon with the location of SFV-positive wild mandrills. Samples collected from SFV-positive wild mandrills are shown in grey. The blue line represents the Ogooué River.

Mandrills in their natural habitats have been reported to be infected with two other retroviruses, simian T-cell leukaemia virus (STLV) [47] and simian immunodeficiency virus (SIV) [42], the strains of each virus were depending on their geographical location. Gabon is separated by the Ogooué River (Figure 3), and studies of cytochrome b polymorphism suggest that the River separates mandrill populations into two different phylogenetic groups: one in northern Gabon and Cameroon and the other in southern Gabon and Congo [52]. 

**Figure 4:**
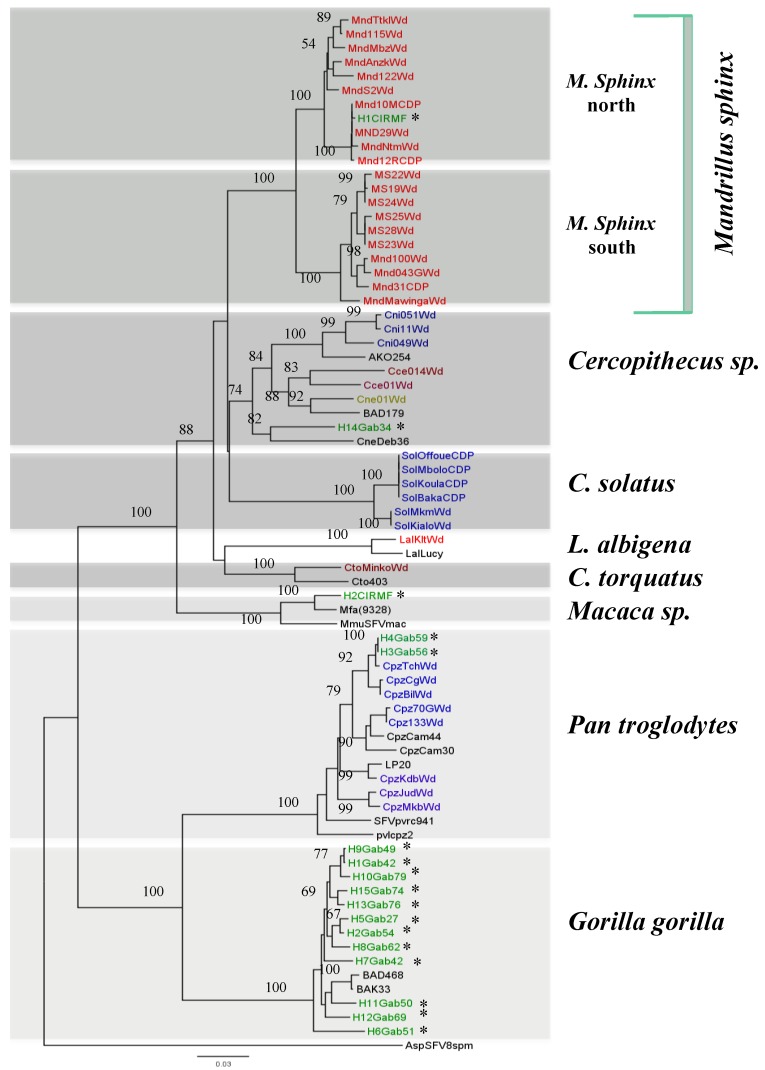
Phylogenetic relations of *integrase* sequences (425 bp) obtained from semi-free-ranging mandrills, wild-born mandrills, wild-born monkeys and apes as well as from SFV-infected humans in Gabon. SFV sequences were aligned with ClustalW (1.81) and edited with Bioedit. Phylogenetic analyses were performed by the Bayesian Markov chain Monte Carlo method implemented in Mr Bayes 3.1 and the Rtrev model. Sequence Asp8SFVsp (from a spider monkey) was included as an out group. Values above the branches are bootstrap values. Non-human primates are indicated by the name of the species (e.g., Mnd, for mandrill), the number of the sample (e.g., 122), followed by Wd (wild) for the origin. (*) indicate SFV sequences obtained from humans, who are also indicated by H, an order number, Gab (for Gabon) and a number.

To confirm the origin of the mandrills in the colony at the CIRMF and to understand the circulation of various strains of SFV and their geographical origin, two studies were conducted. In the first, we amplified and sequenced a portion of the cytochrome b sequence from SFV-infected monkeys in the colony and from mandrills caught in the wild in various regions of Gabon. Two distinct clusters were found, with a perfect correlation between cytochrome b sequence and origin: from north and south of the Ogooué River [22]. The second study was conducted to confirm the hypothesis that mandrills are infected naturally with two different SFV strains. We amplified and sequenced SFV from DNA in blood or tissue samples collected from eight wild mandrills from northern Gabon and seven from the southern part (Figure 3) and compared the sequences with those in our colony. Phylogenetic analysis confirmed that the mandrills in our colony are infected with both SFV strains, from north and south of the Ogooué River (Figure 4). 

SFV has existed in various species of monkey and apes for a long time [2,9,12,34,35,37,38,53]. Mandrills might have been infected with SFV when they had a common ancestor, and the infection has persisted since their separation about 800,000 years ago [52]. As indicated above, our results show that mandrills are also infected with two different strains of SFV, as for SIVmnd [42] and STLV-1 [47]. 

## 3. SFV Infection in Wild-Born Non-Human Primates

**Figure 5:**
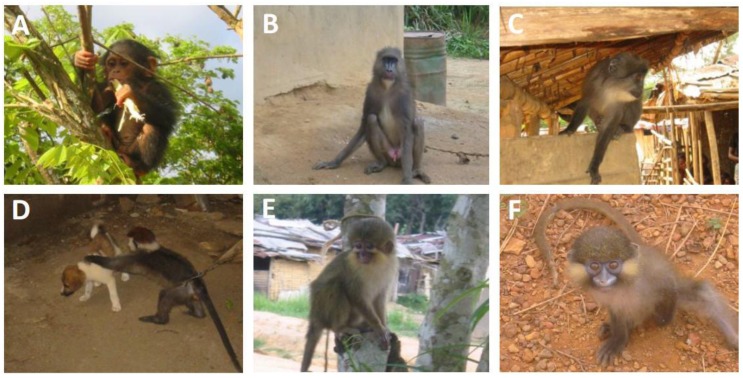
A chimpanzee and monkeys kept in various villages as pets after their parents had been killed by hunters in the forest. (**A**) *Pan* *troglodytes troglodytes* (chimpanzee); (**B**) *Mandrillus sphinx* (mandrill); (**C**) *Cercopithecus solatus* (sun-tailed monkey); (**D**) *Cercocebus torquatus* (red-capped mangabey); (**E**) *Miopithecus ogoouensis* (Gabon talapoin) and (**F**) *Cercopithecus cephus* (moustached monkey).

**Figure 6:**
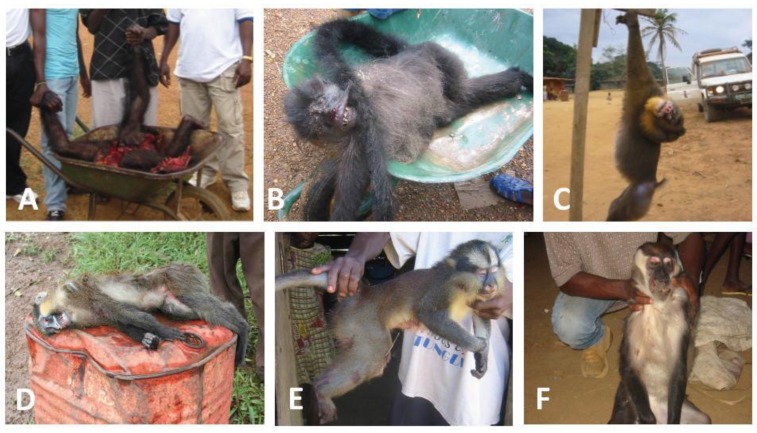
Non-human primates sold as bush meat in villages in rural Gabon. (**A**) *Pan troglodytes troglodytes*; (**B**) *Colobus satana*; (**C**) *Mandrillus sphinx*; (**D**) *Cercopithecus cephus*; (**E**) *Cercopithecus neglectus* and (**F**) *Cercocebus torquatus*.

Although all non-human primates examined to date, including various species of monkey and ape in Africa, were infected with SFV [10,11,12], little is known about SFV infection, transmission and dissemination in natural conditions. To enhance our knowledge of the circulation of SFV in central Africa, we conducted two epidemiological studies in Gabon, where a wide diversity of non-human primates live deep in the rainforest and where hunting monkeys and apes is still frequent, with wide circulation of “bush meat”. The first study was carried out to determine the prevalence of circulating strains of SFV in wild-living mandrills collected as pets, and the second study was carried out on bush meat collected from various markets across the country (Figure 3) [20]. We obtained 273 blood samples from animals kept as pets (Figure 5) after their parents had been killed by hunters in the forest and obtained 211 samples from non-human primates sold as bush meat (Figure 6). Western blot analysis showed antibodies to SFV in 10.8% of plasma samples obtained from animals kept as pets, and PCR showed the presence of SFV in 4.7% of bush meat samples and 9.8% of pets. Interestingly, as seen in Table 1, we identified species-specific SFVs in *Cercopithecus solatus*, *C. nictitans* and *C. cephus*, which have not been reported previously to be infected with SFV (see also Figure 4). Other species, such as *Mandrillus sphinx*, *Pan troglodytes troglodytes*, *Cercopithecus neglectus*, *Cercocebus torquatus* and *Lophocebus albigena*, were also infected with SFV [22]. 

**Table 1:** The presence of SFV in various species of monkeys and apes collected in Gabon, central Africa.

Species	Common name	SFV	
		Serology	PCR
*Cercopithecus neglectus*	De Brazza guenon	NA	+
*Cercopithecus solatus*	Sun-tailed monkey	+	+
*Cercopithecus cephus*	Red-eared guenon	NA	+
*Cercopithecus nictitans*	Greater white-nosed monkey	NA	+
*Mandrillus sphinx*	Mandrill	+	+
*Pan troglodytes troglodytes*	Central African chimpanzee	+	+
*Cercocebus torquatus*	Red-capped mangabey	+	+
*Lophocebus albigena*	Grey-cheeked mangabey	+	+

NA: not available

The estimated prevalence of SFV in the wild, as determined in pets collected at a young age and in bush meat, was much lower than that in mandrills living at the CIRMF colony. Although it has been reported that 50%–100% of monkeys are infected with SFV, most of the studies involved monkeys born in the wild but living in semi-free conditions, such as zoos, national parks and breeding colonies, like our free-living mandrills in the colony at the CIRMF, in which we found a prevalence of 50%–100% depending on the age group, with the lowest prevalence in juvenile monkeys [20]. A seroprevalence of 89.5% was also found in a small macaque population (mostly adults) living in a temple in Bali, Indonesia, with a higher prevalence in adults than in juveniles [23]. Thus, young monkeys are less frequently infected than older ones. In the our study in wild monkeys and apes in Gabon, most samples were collected from pet monkeys captured as juveniles, which may explain in part the low prevalence in these samples.

## 4. SFV Cross-Species Transmission to Humans in Gabon

Zoonotic transmission of SFV from various non-human primates to occupationally exposed people, such as zookeepers, veterinarians and personnel of animal care facilities, is well documented [25,26], and bites from adult non-human primates are presumed to be the major risk factor for viral acquisition. Acquired SFV infection has also been reported in hunters in the rainforest of south Cameroon and in people living in close contact with macaques in various Asian countries [18,19,54]. In Cameroon, 24% of people who were bitten while hunting apes were infected with SFV [19]. Neither signs of infection-associated disease in humans nor human-to-human transmission of SFV have, however, been documented [19,25,26,27]. 

**Figure 7:**
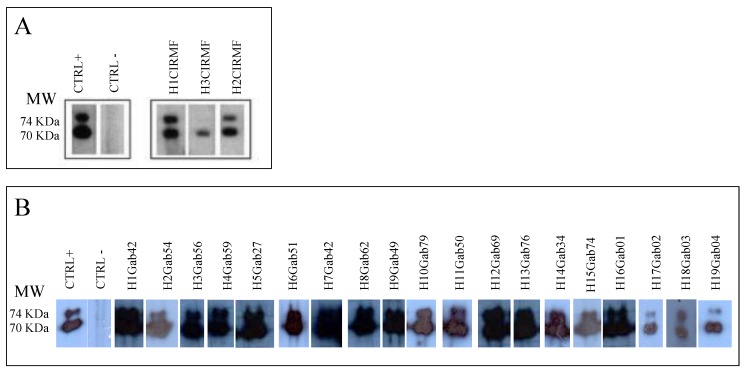
SFV-specific antibodies detected by western blot in plasma samples obtained from humans bitten by non-human primates in Gabon. Seropositivity was defined by the presence of reactivity to the Gag doublet of 70 kDa and 74 kDa as shown for positive controls (CTRL+); seronegativity was defined as no bands of the Gag doublet observed by western blot, as in the negative control (CTRL–). Reactivity with a single band in the 70- to 74-kDa molecular mass range was considered indeterminate. (**A**) Western blot pattern of the three people bitten by non-human primates at the CIRMF; (**B**) western blot pattern of individuals bitten by wild non-human primates in various regions of rural Gabon.

Before 2010, no studies had been conducted on the transmission of SFV in Gabon. We investigated interspecies transmission of SFVs from non-human primates to humans in this country, where exposure to non-human primates is high. We first evaluated the possible transmission of SFV from mandrills living in our colony at the CIRMF to humans by examining 20 people occupationally exposed as caretakers or veterinarians at the Primatology Centre. The mean duration of exposure was 12 years. Two of these people were found to be SFV seropositive by western blotting [20], while the results for one were indeterminate (Figure 7A). The two seropositive individuals were also positive by PCR. The first person had been bitten by a chimpanzee on a finger in 1996 and by a mandrill on a shoulder in the same year. The second person recalled a bite on a finger by an unknown monkey in 1985. Phylogenetic analysis of the *integrase* fragments (Figure 4) showed that the viruses from the first individual (H1CIRMF) and from mandrills were almost identical, with only one base difference (99.7% nucleotide identity). Phylogenetic analysis of the SFV obtained from the second person (H2CIRMF) showed that the virus was located in the clade of Asian SFVs (bootstrap, 96%) and clustered with *Macaca fascicularis* (Figure 4). Only one molecular demonstration of SFV interspecies transmission has previously been reported, in a zoo worker bitten by a chimpanzee [17]. Although the person infected with the mandrill virus in our study had also been bitten by a chimpanzee, we were unable to detect any chimpanzee SFV sequence in his PBMCs. “Dual” risks with only one virus detectable by PCR were also reported in hunters in south Cameroon [19]. Co-infection with two different simian viruses was reported recently in chimpanzees, which were infected not only with their own chimpanzee SFV but also with a *Colobus* strain [55].

**Figure 8:**
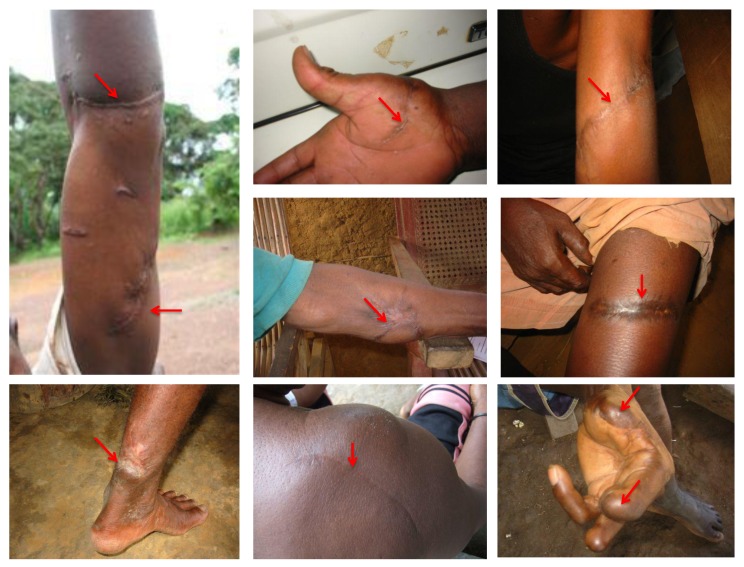
SFV-infected individuals with scars and lesions (red arrow) caused by bites from wild non-human primates during hunting.

In the second study, we collected samples from 78 people (10 women, 59 men and 9 children) who had received severe bites or scratches from non-human primates while hunting or playing with pets (Figure 8) in various regions in the tropical forest. Antibodies against SFV were detected in 24% of samples (Figure 7B). The SFV *integrase* fragment was detected by PCR in 15 DNA samples from the 19 seropositive people and then sequenced. Twelve of the DNA samples were from people who had been severely bitten by gorillas, two from people bitten by chimpanzees and one from a person who had been bitten by an unspecified *Cercopithecus* monkey. As seen in Figure 4, in all cases, there was a perfect match between the history of the contact with a given species and the simian viral sequence found in the infected person [22]. The sequence obtained from the person who had been bitten by an unknown *Cercopithecus* genus clustered with the SFV strain obtained from a De Brazza monkey (Figure 4). 

Our results for SFV in humans are similar to those of Calattini *et al.* [19] and Betsem *et al.* [19,21] in rural Cameroon, who showed a prevalence of 24.1% and 18.6%, respectively, among people who had had contact with gorillas or chimpanzees. The two cohorts are similar and from similar geographical areas of central Africa. Calattini *et al.* [19] also showed that transmission of SFV from non-human primates to humans is more likely after a deep bite and specifically by an ape. 

To evaluate the genetic variation of SFV *in vivo*, we investigated the virus population in one mandrill at an interval of 10 years and also studied the genetic variation of the virus after transmission to a human through a severe bite. We studied several clones obtained in a single sample from the mandrill on the day the person was bitten, clones from the same animal 10 years later and clones from the bitten person 10 years after the bite. Comparative sequence analysis showed strong nucleotide sequence similarity. The clones from the mandrill were identical; the clones in the two mandrill samples and the human samples differed only slightly, with a divergence of one or two bases [20]. We thus observed high stability of SFV over time, with neither genetic drift nor the presence of quasi-species. 

## 5. Conclusion and Remaining Questions

In our studies in Gabon, we found that SFV is highly endemic in mandrills and also in other monkey species and that the virus can be transmitted to humans. Our findings suggest that cross-species transmission of SFV is widespread in central Africa, especially in villages and settlements in lowland forest regions like those in Cameroon, Gabon, Equatorial Guinea, Congo and the Central African Republic, where hunting for bush meat is frequent. Hunting has increased in these regions due to a combination of urban demand for bush meat and easier access to non-human primate habitats via logging roads, and this has increased the frequency of human exposure to non-human primate retroviruses. Apetrei and Marx [56] pointed out that human exposure to retroviruses through hunting and butchering is ancient in central and west Africa, whereas the AIDS epidemic emerged only in the second half of the 20th century. Thus, certain factors intervened in the spread of SIV and its emergence as HIV in human populations, which might include deforestation, increased urbanization and travel and increased unsafe injections and transfusions, which might promote viral adaptation through serial passage or favour adaptation by other mechanisms, such as recombination. None of these theories is proven, and it has been shown that experimental or accidental transmission of SIVs to different species is often cleared by the new host. Furthermore, serial injections of SIV are needed to increase its pathogenicity in a new host [57]. In the case of SFV, it has been shown that foamy viruses are not pathogenic in naturally or experimentally infected hosts. In our studies in Gabon and in other studies in central Africa, no pathological manifestations were found after SFV infection of either mandrills or humans. The two SFV-infected humans at the Primate Centre are healthy and show no clinical signs of retroviral infection 15 years after the bites. Although it is difficult to determine what clinical and biological examinations were undertaken for hunters in rural areas of Gabon, no pathological manifestations were found in the 19 seropositive individuals, even several decades after the bites. In the survey we conducted two years after the SFV-infected hunters were found to be positive, all the hunters and their family members were healthy and showed no clinical signs of retroviral infection. The SFV-infected individuals are followed up clinically each year. At the most recent one, in November 2012, all were healthy. Thus, we can assume that, as for accidental transmission of SIV to humans (see above), a single infection with SFV cannot induce pathological manifestations, and therefore humans are not yet susceptible to diseases associated with SFV infection. Serial passage (transmission) of the virus between humans would be needed to increase the pathogenicity of the virus and induce illness. 

Furthermore, in infections that result in zoonoses, like rabies, haemorrhagic fever and Ebola, the animal source (which is generally a chimpanzee or gorilla in the case of Ebola) is often susceptible to the disease. In the case of SFV, however, no diseases are clearly associated in infected animals. Thus, individuals infected with SFV do not develop any associated disease, and we should therefore be careful in defining SFV infection as a “zoonosis”. Other factors, such as recombination or the immune status of the infected human, could, however, play a role in the transmission, dissemination and induction of SVF-associated diseases in human populations and should be evaluated. Recently Feeroz *et al.* [58] reported an interesting study on the population dynamics of rhesus macaques infected with foamy virus in Bangladesh. They showed that recombinant SFVs are common in Asian macaques and that they may be co-infected with more than one SFV. This study illustrates the interplay between host ecology and viral evolution that can lead to the emergence of new viruses of greater pathogenicity. Similar studies could be carried out in Africa to better understand the role and effects of recombinant strains in the development of disease. Another work published by Switzer *et al.* [50] in which they conducted epidemiological studies on urban commercial sex workers, patients with sexually transmitted diseases and blood donors in the Democratic Republic of the Congo and found one commercial sex worker and one blood donor to be seropositive for both SFV and HIV-1. These represent the first cases of dual SFV and HIV infection. The prevalence of HIV is high in Gabon [59] and in other central African countries, and access to antiretroviral therapy is limited. In view of the study of Switzer *et al.*, it will be important to define the clinical consequences of concomitant infection with a virus that induces immunosuppression in humans, such as HIV, and to determine the risk for SFV transmission by blood transfusion and by humans, such as by sexual and mother-to-child transmission. Further investigations of people infected with SFV are needed in both central Africa and South-East Asia to determine inter-human transmission in familial studies and the morbidity and mortality that might be associated with this “zoonotic” infection, especially in people infected with HIV. 
